# Complete Androgen Insensitivity Syndrome (CAIS) Genetic Counseling: Navigating Germline Mosaicism Concerns

**DOI:** 10.1155/crig/1224893

**Published:** 2026-04-11

**Authors:** Lauren M. Iacono, Paul A. Levy, Tamar G. Baer

**Affiliations:** ^1^ Department of Pediatrics, University of Vermont Children’s Hospital, Larner College of Medicine at the University of Vermont, Burlington, Vermont, USA; ^2^ Department of Pediatrics, Children’s Hospital at Montefiore, Albert Einstein College of Medicine, Bronx, New York, USA, yu.edu

**Keywords:** androgen receptor gene mutation, androgen receptor genetic variant, complete androgen insensitivity syndrome, genetic counseling, germline mosaicism

## Abstract

Complete androgen insensitivity syndrome (CAIS) is caused by pathogenic variants in the androgen receptor (AR) gene that lead to a phenotypically female appearance in XY individuals. It is almost always inherited as an X‐linked recessive condition. Here, we present two sisters with different clinical courses. AR gene sequencing revealed identical hemizygous pathogenic variants in both sisters but not in the mother. This rare occurrence of germline mosaicism is the first described in CAIS. Germline mosaicism should be considered when “*de novo*” AR gene variants are identified. Despite the low recurrence risk, counseling would be beneficial to families so they can make well‐informed prenatal and reproductive plans.

## 1. Introduction

Androgen insensitivity syndrome (AIS) is a complex condition and can manifest as three broad phenotypes. Each has a 46, XY genotype, though insensitivity varies phenotypically from complete AIS (CAIS) to partial AIS (PAIS) to mild AIS (MAIS). AIS has an overall prevalence of 2–5 per 100,000 births [1]. The prevalence of CAIS proven through molecular diagnosis is estimated to occur in 1 in 20,400–99,100 genetic males [2]. The cause of AIS is a pathogenic variant in the androgen receptor (AR) gene. The malfunctioning receptor results in insensitivity to androgens and development of a female phenotype in XY individuals [3, 4]. Androgen insensitivity is inherited as an X‐linked recessive condition. XX individuals who inherit the variant are carriers, whereas XY individuals who inherit the pathogenic variant are affected [4, 5].

Pathogenic variants in the AR gene lead to loss of function of the AR [2]. In the complete form of AIS, the variant leads to complete disruption of the AR and hormonal resistance to testosterone and dihydrotestosterone (DHT) [6]. During normal fetal development, the XY zygote directs the bipotential gonads to become testes, which then produce active androgens and stimulate target cells leading to appropriate male sexual differentiation [4]. However, in CAIS, although the XY zygote directs the bipotential gonads to become testes, the AR is resistant to the androgens produced, resulting in a female phenotype [3, 4].

CAIS may be diagnosed in both adolescence and in infancy depending on presentation. In adolescence, affected individuals have typical breast development due to aromatization of circulating androgens to estrogen and development of external female genitalia due to androgen insensitivity. Their presenting complaint is often primary amenorrhea due to the absence of internal female structures. Internally, these individuals have bilateral testes in the lower abdomen or inguinal canal [3, 6]. In infancy, a phenotypically appearing female with CAIS may present with bilateral inguinal hernias or with labial swelling. Often when these hernias are repaired, they are noted to in fact be testes. Due to intact sensitivity to anti‐Müllerian hormone (AMH) produced by the testicular Sertoli cells, individuals with CAIS will also have a shortened vagina and absence of the uterus, cervix, and proximal vagina [3, 4].

The biochemical profile of individuals with CAIS may vary based on age of diagnosis and if gonads remain intact. In those with CAIS and intact gonads, testosterone is produced in excess due to negative feedback at the pituitary level and peripherally aromatized into estrogen. In addition, there is luteinizing hormone (LH)‐induced direct secretion of testicular estrogen. Together, both factors lead to higher‐than‐normal estrogen levels in males but lower than those in normal XX females [3, 4, 7]. LH is inappropriately elevated, while follicle stimulating hormone (FSH) and inhibin levels are within the normal range. When detected in infancy, the gonadotropin and testosterone concentration patterns are less suggestive of hormone resistance compared with those unaffected as even infants without CAIS have a transient elevation in LH and testosterone levels, called mini puberty. However, in older children and adolescents with CAIS, there is marked elevation in LH levels and concurrent normal to high levels of testosterone compared with those unaffected, indicating that the hypothalamic–pituitary axis is overall unresponsive to the inhibitory feedback of circulating testosterone [3, 7, 8]. Infants with CAIS also have higher concentrations of AMH compared with normal XY males [3, 6].

An AR pathogenic variant is identified in more than 95% of the patients with CAIS. In 70% of the cases, the AR pathogenic variant is transmitted in an X‐linked pattern through maternal carriers; however, *de novo* variants can occur in up to 30% of the cases [9, 10]. Typically, if a family has more than one affected XY individual, the mother of the affected child is an obligate heterozygote or carrier [1, 5]. The recurrence risk of the same de *novo* variant in a sibling is estimated to be less than 1%. Therefore, if there is more than one child affected and the pathogenic variant cannot be detected in maternal leukocyte DNA, germline mosaicism is most likely [1]. Here, we have such a case of two affected sisters found to have identical pathogenic variants in the AR gene without the variant present in their mother’s leukocyte DNA, demonstrating germline mosaicism.

## 2. Case Presentation

The two sisters in our case are the only children born to nonconsanguineous parents of Dominican descent. Their parents reported no medical problems. The sisters have 3 maternal aunts, all with children. They have 1 paternal uncle and 1 aunt, both who have children. There are no family members reported with amenorrhea, infertility, genital ambiguity, or similar history of presentation to the two sisters. The younger sister was referred to pediatric endocrinology by her pediatrician soon after arrival from the Dominical Republic at age 11. She was born in Santo Domingo, and there was no report of atypical genitalia at birth. At around age 1‐2 years, the mother was informed that the child had bilateral inguinal hernias, with a plan to operate once she was older. At 8 years of age, she presented with significant abdominal pain prompting surgical correction of the hernias and, as per her mother, two small “masses” that appeared to have gonadal tissue were removed. Chromosome analysis was performed showing a 46, XY karyotype, which prompted referral to endocrinology. On presentation at age 11 years, she had no dysmorphisms. Breast exam revealed adipomastia with no palpable breast buds and immature nipples. External genitourinary exam showed no pubic hair (Tanner stage I) with normal appearing labia majora, minora and clitoral hood, and separate urethral and vaginal openings. An ultrasound showed absence of uterus and ovaries. Laboratory evaluation was notable for elevated gonadotropin levels, with low testosterone and estradiol levels (Table 1), consistent with gonadectomy and hypergonadotropic hypogonadism. Her AMH and inhibin B levels were also low, with normal 17‐hydroxyprogesterone, 17‐hydroxypregnenalone, deoxycorticosterone (DCO), 11‐deoxycortisol, and dehydroepiandrosterone–sulfate (DHEA–S) levels (Table 1). She is currently being treated with estrogen replacement therapy for induction of puberty and bone health, given gonadectomy.

**TABLE 1:** Laboratory results for the 11‐year‐old patient on presentation, status post gonadectomy.

Laboratory test	Result	Reference range (female)
Testosterone	8	**< 40** ng/mL
Luteinizing hormone	23.66 (H)	**≤ 0.15** mIU/mL
Follicle stimulating hormone	> 86.00 (H)	**0.87–9.16** mIU/mL
Estradiol	< 4 (L)	**< 16** pg/mL
Inhibin B	< 10 (L)	**< 86** pg/mL
17‐Hydroxyprogesterone	13	**≤ 196** ng/dL
Dehydroepiandrosterone**–**sulfate	238	**35–430** mcg/dL
Anti‐Müllerian hormone	0.01 (L)	**> 1.02** ng/mL

*Note*: Bold is the reference range.

The older sister presented with primary amenorrhea. At 15 years of age, while in the Dominican Republic, an ultrasound showed absence of ovaries and uterus. Chart notes indicate she was being evaluated for Mayer–Rokitansky–Küster–Hauser syndrome. On presentation to our endocrinology team at 19 years of age, her exam was notable for Tanner stage V breasts, Tanner stage II pubic hair that was sparse and light in color, and no axillary hair. On genitourinary exam, she had normal labia majora, labia minora, and clitoris. Her vaginal length was short at 3.5 cm in depth, and she had a thickened hymen. She had separate vaginal and urethral openings. MRI showed a blind‐ending vagina, absent uterus. Also noted on ultrasound were pelvic testicles with bilateral paratesticular cysts and a left testicular Sertoli cell adenoma. Her laboratory evaluation revealed a 46, XY karyotype, elevated testosterone of 609 ng/dL (female range: 2–45 ng/dL), inhibin B 550 pg/mL (female range: 47–308 pg/mL), and estradiol 14 pg/mL (female range: 39–440 pg/mL). Her 11‐deoxycortisol, DOC, DHEA–S, 17‐hydroxyprogesterone, and 17‐hydroxyprenenalone levels were all within the normal range (Table 2). With multidisciplinary team counseling and shared decision‐making, she deferred gonadectomy and will continue to follow with our team for monitoring.

**TABLE 2:** Laboratory results for the 19‐year‐old patient on presentation, with gonads retained.

Laboratory test	Result	Reference range (female)
Testosterone	609 (H)	**2–45** ng/dL
Luteinizing hormone	13.9	**0.10–14.7** mIU/mL
Follicle stimulating hormone	2.9	**0.64–10.9** mIU/mL
Estradiol	14 (L)	**39–440** pg/mL
Inhibin B	550 (H)	**47–308** pg/mL
17‐Hydroxyprogesterone	39	**33–583** ng/dL
Dehydroepiandrosterone**–**sulfate	302	**35–430** mcg/dL
Dihydrotestosterone	26 (H)	**< 20** ng/dL
Anti‐Müllerian hormone	> 240 (H)	**1.02–14.63** ng/mL

*Note*: Bold is the reference range.

Genetic testing results showed that both sisters to have identical pathogenic variants in the AR gene, c.2678C > T (p.Pro893Leu), with hemizygosity. Given that AIS is an X‐linked condition, their mother was also tested. Her results, however, did not show this AR gene variant, suggesting a possible germline (or gonadal) mosaicism as the explanation for the two affected children.

## 3. Discussion

Mosaicism in gonadal cells has been recognized as an important mechanism in genetic disorders [9]. Mosaicism is the occurrence two or more genomes derived from a single zygote in one individual and may be classified as germline or somatic. Germline mosaicism is defined as a cell line restricted to the gonads that is not present in other tissues and can be passed on to the offspring. These variants selectively occur postzygotically in germ cells within the gonads. This mosaicism may arise during meiosis, when germ cells are formed postzygotically, or throughout the human lifespan by spontaneous mutagenesis. In either instance, this mosaicism can be later passed to the offspring as inherited germline variations, while the parent may remain phenotypically normal [10].

Conversely, somatic mosaicism occurs postzygotically in the somatic cells and can occur at any developmental stage or even in adult tissue. It does not originate in the germ cells, and in human disease, occurs more frequently than germline variants. These postzygotic variants can manifest throughout the entire body if the variant occurs early in development or can be confined to specific body regions. Mosaicism in one individual can occur in both germline and somatic cells, depending on variant developmental timing. The adult with both germline and somatic mosaicism has the risk of not only transmitting to their offspring but also the risk of having the disease themselves [10].

When pedigree analysis does not match the known inheritance pattern, this is likely an indication of germline mosaicism, especially if two or more offspring are affected [11]. For example, if a known autosomal dominant condition is identified in an offspring but neither parent is found to have the pathogenic variant, then germline mosaicism is most likely the cause. The offspring subsequently carries this germline variant, which is present in all of their cells (both germline and somatic cells). Confirmatory testing of the germline is challenging as this would require invasive tissue sampling of the ovaries. Some well‐known genetic disorders prone to germline mosaicism include X‐linked Duchenne muscular dystrophy, autosomal dominant osteogenesis imperfecta, and some aneuploidy conditions, such as in Trisomy 21, where about 5% or more of the cases are due to a germline pathogenic variant [10].

*De novo* variants are changes that occur in an offspring that are not detected in the genome of either parent. There are three possible patterns in the recurrence of *de novo* structural and/or combined numeric and structural rearrangements in siblings: recurrence by chance, germline mosaicism, and low‐level somatic‐germline mosaicism [12]. The chance of recurrence risk of the same pathogenic variant in two siblings when the variant is absent in the parental genome is estimated to be less than 1% for *de novo* variants [13]. Therefore, it would be highly unlikely that two or more siblings would be affected due to a new variant in the same gene within the same family [11] and so germline mosaicism is important to consider [9]. Counseling for germline mosaicism is important, as it may influence prenatal and reproductive decisions [13].

Seventy percent of the cases of AIS are transmitted in an X‐linked recessive manner through the maternal carrier. However, in 30% of the cases, the variants may arise *de novo* [14]. Because the recurrence risk for *de novo* variants is infrequent, the presentation of our two siblings strongly suggested that the inheritance pattern of the pathogenic variant is due to germline mosaicism [13]. The identical pathogenic variants in the AR gene in both sisters in the absence of a maternal variant have not been reported in the literature previously. There have been reports in the literature of affected probands without maternal somatic variants, but most of these are individual *de novo* cases and none report siblings with identical pathogenic variants. Kohler et al. reported on six unrelated families with *de novo* somatic pathogenic variants in the AR gene. Four of these presented with atypical genitalia at birth (diagnosed as PAIS), whereas two had normal female genitalia (CAIS). The seventh patient had AR gene sequencing, which identified a novel hemizygous germline variant, although the mother presented a sequence pattern compatible with a somatic mosaicism of the pathogenic variant [14].

## 4. Conclusion

Mosaic variation is a biological process that has wide‐ranging implications in regard to variant selection, development, and disease [10]. Our case highlights the importance of considering germline mosaicism in affected siblings with a genetic condition when neither parent demonstrates the phenotype nor is noted to have the pathogenic variant [15]. Although the inheritance of genetic variants through germline mosaicism has been describe in other disorders [12], this is the first case of siblings described with CAIS. Investigation may be indicated in *de novo* variants of children of an unaffected parent(s), even in families with a single affected child, as maternal germline mosaicism or additional female (XX) siblings with carrier status may be revealed [15]. Unexpected recurrences may occur, as seen here with multiple affected children who harbor the same apparently *de novo* pathogenic variant. The birth of a single child with a genetic disease, especially when severe, can pose social, economic, and psychosocial challenges. Therefore, families having had a child with an apparently *de novo* pathogenic variant can benefit from transparency and well‐informed prenatal and reproductive counseling [13].

## Funding

The authors have nothing to report.

## Ethics Statement

Written informed consent was obtained from participants (or their parents/legal guardians/next‐of‐kin) for publication of the details of their medical case and any accompanying images.

## Conflicts of Interest

The authors declare no conflicts of interest.

## Data Availability

Data sharing is not applicable to this article as no datasets were generated or analyzed during the current study.
